# Clinical Characteristics of COVID-19-Infected Cancer Patients in Pakistan: Differences Between Survivors and Non-Survivors

**DOI:** 10.3389/fonc.2021.655634

**Published:** 2021-05-20

**Authors:** Kashif Asghar, Muhammad Abu Bakar, Muhammad Junaid Akram, Asim Farooq, Kashif Siddique, Iftikhar Ali Rana, Jamshed Ali, Muhammad Usman Rashid, Ashraf Ali Khan, Asif Loya

**Affiliations:** ^1^ Department of Basic Sciences Research, Shaukat Khanum Memorial Cancer Hospital and Research Centre, Lahore, Pakistan; ^2^ Department of Cancer Registry and Clinical Data Management, Shaukat Khanum Memorial Cancer Hospital and Research Centre, Lahore, Pakistan; ^3^ Department of Internal Medicine (Pulmonology), Shaukat Khanum Memorial Cancer Hospital and Research Centre, Lahore, Pakistan; ^4^ Department of Clinical Research, Shaukat Khanum Memorial Cancer Hospital and Research Centre, Lahore, Pakistan; ^5^ Department of Radiology, Shaukat Khanum Memorial Cancer Hospital and Research Centre, Lahore, Pakistan; ^6^ Department of Pathology, Shaukat Khanum Memorial Cancer Hospital and Research Centre, Lahore, Pakistan; ^7^ Department of Medical Oncology, Shaukat Khanum Memorial Cancer Hospital and Research Centre, Peshawar, Pakistan; ^8^ Department of Internal Medicine (Infectious Diseases), Shaukat Khanum Memorial Cancer Hospital and Research Centre, Lahore, Pakistan

**Keywords:** COVID-19, cancer, survivors, Pakistan, non-survivors

## Abstract

**Background:**

Cancer patients are considered as highly vulnerable individuals in the current COVID-19 pandemic. We studied the clinical characteristics of survivor and non-survivor COVID-19-infected cancer patients in Pakistan.

**Patients and Methods:**

We did a retrospective study of 70 cancer patients with PCR-confirmed COVID-19 infection from Shaukat Khanum Memorial Cancer Hospital and Research Centre, Lahore and Peshawar, Pakistan between April 13 and July 09, 2020. These patients were discharged from the hospital or had died by July 09, 2020. Clinical, pathological and radiological characteristics were compared between survivors and non-survivors by fisher’s exact test and chi-square test. Univariable and multivariable logistic regression models were performed to explore the risk factors of mortality.

**Results:**

Seventy cancer patients with SARS-CoV-2 infection were enrolled and the majority were males 38 (54.3%). 57 (81.4%) had solid tumors and 13 (18.6%) had hematological malignancies. Dyspnea (44 cases) was the most common symptom (62.9%). Complications were reported in 51 (72.9%) patients during the course of disease. 19 (27.1%) patients were admitted to an intensive care unit (ICU). A significant increase in the C-reactive protein level and neutrophil count was observed in the deceased patients as compared to the surviving patients. D-dimer values of ≥0.2 mg/L were significantly associated with mortality (*P*=0.01). We identified two independent risk factors associated with death, ICU admission (*P*=0.007) and D-dimer (*P*=0.003).

**Conclusion:**

Pakistani cancer patients with COVID-19 infection reported poor prognosis. Intensive surveillance of clinicopathological characteristics of cancer patients infected with COVID-19 especially D-dimer values may play a pivotal role in the outcome of the disease.

## Introduction

The coronavirus disease of 2019 (COVID-19) is caused by severe acute respiratory syndrome coronavirus 2 (SARS-CoV-2) (1). The virus was first detected in December 2019, in Wuhan, China (2). The whole world including Pakistan is experiencing COVID-19 outbreaks (3). The World Health Organization (WHO) declared the COVID-19 outbreak a pandemic on March 11, 2020 (4). As of March 11, 2021, 117,160,237 cases have been confirmed worldwide, with 2,598,892 deaths (5). In Pakistan 597,497 COVID-19, cases and 13,377 deaths have been confirmed, as of March 12, 2021 (6).

Epidemiological data have indicated that cancer patients are more susceptible to the COVID-19 infection (7–11). SARS-CoV-2 infection affects the functioning of cardiovascular, renal, hepatic, pancreatic and gastrointestinal systems (8). Furthermore, the virus has the ability to invade and cause neural disease in the central and peripheral nervous system (8, 12). There is an increased risk of opportunistic infections in cancer patients due to their impaired immunity, poor nutrition and post cancer therapy associated side effects (7). Liang et al. demonstrated that cancer patients with COVID-19 had poorer outcomes (13). Zhang et al. also reported that cancer patients with COVID-19 showed poor prognoses (14). Comprehensive data published by Yang and colleagues concluded that patients with cancer and COVID-19 are a high-risk population with an increased mortality rate (7).

Each year around 200,000 new cancer cases are being diagnosed in Pakistan (15) and approximately, 45,000 cancer patients come to the Shaukat Khanum Memorial Cancer Hospital and Research Centre (SKMCH & RC). SKMCH & RC is a state-of-the-art charitable hospital where 75% of patients diagnosed with cancer are treated free of charge (16). The hospital also accepts cancer patients from Afghanistan.

We analyzed the data from cancer patients with SARS-CoV-2 infection who were admitted to SKMCH & RC. Here, we report the systematically characterized clinical features of survivors and non-survivors COVID-19-infected cancer patients in Pakistan, in an attempt to define the nature of the aggravating factors caused by COVID-19 infection.

## Methods

### Study Design and Participants

The current study was conducted at SKMCH & RC Lahore and Peshawar, Pakistan. This is a retrospective cohort study. The first cases of SARS-CoV-2 were identified in Pakistan in February 2020 (3). Immediately after the COVID-19 outbreak, SKMCH & RC Lahore and Peshawar initiated the diagnosis and treatment of SARS-CoV-2 infected cancer patients. COVID-19 patients were diagnosed based on the guidelines provided by WHO (17). All enrolled patients had previously been diagnosed with cancer. We included all patients (aged ≥ 18 years) with pathological diagnoses of any malignant solid or hematological malignancies along with PCR-confirmation of SARS-CoV-2 infection. We aimed to investigate the clinical characteristics of survivors and non-survivors COVID-19-infected cancer patients in Pakistan. All patients fulfilling the eligibility criteria were included in the study. The study cutoff date was July 09, 2020. The institutional review board (IRB) of SKMCH & RC approved the current study (IRB-EX-20-04-20-01). IRB granted the waiver of the written informed consent from study participants.

### Data Collection

The information about the demographic data, clinicopathological characteristics, laboratory findings and chest X-ray examinations were acquired from pathology and radiology reports from our hospital medical records’ system. Data about gender, age, comorbidities (diabetes, hypertension, cardiac disease, chronic pulmonary disease, chronic kidney disease and chronic liver disease), cancer history (cancer type, stage and treatment), vital signs (temperature, heart rate, respiratory rate, blood oxygen saturation), body mass index and symptoms (dyspnea, cough, fever, muscle ache, sore throat, diarrhea, vomiting, chills, abdominal pain and headache) and pathology lab tests (white blood cells, neutrophils, lymphocytes, platelets, red blood cells, hemoglobin, creatinine, blood urea nitrogen, lactate dehydrogenase, lactate, pH, PCO2, PO2, HCO3, O2 saturation, alanine transaminase (ALT), aspartate transaminase (AST), albumin, globulin, gamma-glutamyl transpeptidase (GGT), alkaline phosphatase, C-reactive protein, ferritin and D-dimer) were all obtained at the time of diagnosis of COVID-19 in cancer patients. According to the TNM staging system, cancers were defined as stages I, II, III and IV. We also collected the information about treatments for cancer patients with COVID-19 (intravenous antibiotics, antiviral therapy, immunotherapy, anthelmintic agent, montelukast, hydroxychloroquine, zinc/vitamin-C, continuous renal replacement therapy, oxygen therapy and mechanical ventilation), complications and outcomes during admission to hospital.

### Statistical Analysis

We hypothesized that differences exist in demographic, clinical, and laboratory characteristics, treatments, and cancer history between survivors and non-survivors of COVID-19 with cancer. Quantitative variables were presented as medians/mean (range: minimum-maximum/standard deviation), and qualitative variables were presented by frequencies and percentages. The independent t-test, Mann-Whitney U test, Chi-square test, and Fisher‘s exact test were applied to analyze the differences between groups according to the type of data. Kaplan-Meier analysis (log-rank test) was used to check the survival difference. Risk factors associated with death and their odds ratios (ORs) were analyzed by the univariable logistic regression model. Variable selection for multivariable analysis was based on significance from the univariable logistic regression analysis (p<0·05). We used IBM SPSS Statistics 20.0 software for statistical analysis. The tests we used were all two-sided with less than 5% type I error. The differences between groups were considered to be significant when the *P*-value was less than 0·05.

## Results

From April 13 to July 09, 2020, 13,692 patients were diagnosed with COVID-19 in an electronic medical record system from SKMCH&RC, Pakistan. Among 13,692 patients, 443 (3.23%) had cancer. 81 patients with COVID-19 symptoms were admitted at SKMCH&RC. After excluding 11 patients (under the age of 18 years), we included only 70 (15.80%) out of 443 hospital admitted cancer patients as shown in (**Supplementary Figure 1**). None of the 70 patients was lost to follow-up during the hospital admission. Of the 70 cancer patients 30 (42.9%) had died as of July 9, 2020, including, 22 (73.33%) patients who belonged to the Punjab province. The primary cause of death was COVID-19 infection in these patients. 38 (54.3%) patients were male, 32 (45.7%) patients were female, and overall follow-up for all the patients was 88 days. The overall mean age was 50.27 ± 16.61 (**Table 1**). Approximately two-thirds of the patients (72.85%) were admitted to the hospital within 24 hours after diagnosis. 67 (95.71%) of 70 patients had symptoms during the course of COVID-19.

**Table 1:** Demographics and baseline characteristics of patients with cancer and COVID-19.

	Total (n=70)	Alive (n=40, 57.1%)	Dead (n=30, 42.9%)	*P*-value
**Demographics**				
**Age (years)**				0.18
Mean ± SD	50.27 ± 16.61	47.98 ± 15.98	53.33 ± 17.20	
**Sex**				0.18
Male	38 (54.3%)	19 (47.5)	19 (63.3)	
Female	32 (45.7%)	21 (52.5)	11 (36.7)	
**Geographic origin**				0.20
Punjab	53 (75.7)	31 (77.5)	22 (73.3)	
Khyber Pakhtunkhwa	10 (14.3)	4 (10.0)	6 (20.0)	
Federally Administered Tribal Area	1 (1.4)	0 (0.0)	1 (3.3)	
Afghanistan	6 (8.6)	5 (12.5)	1 (3.3)	
**Clinical characteristics and outcomes**				
**Comorbidities**				
Diabetes	9 (12.9)	5 (12.5)	4 (13.3)	1.00
Hypertension	14 (20.0)	8 (20.0)	6 (20.0)	1.00
Chronic pulmonary disease	8 (11.4)	5 (12.5)	3 (10.0)	1.00
Chronic kidney disease	7 (10.0)	3 (7.5)	4 (13.3)	0.45
Coronary heart disease	3 (4.3)	2 (5.0)	1 (3.3)	1.00
Chronic liver disease	3 (4.3)	2 (5.0)	1 (3.3)	1.00
**Symptoms**				
Dyspnea	44 (62.9)	20 (50.0)	24 (80.0)	0.01
Cough	40 (57.1)	25 (62.5)	15 (50.0)	0.29
Fever	24 (34.3)	15 (37.5)	9 (30.0)	0.51
Muscle ache	22 (31.4)	14 (35.0)	8 (26.7)	0.45
Sore throat	17 (24.3)	11 (27.5)	6 (20.0)	0.46
Diarrhea	9 (12.9)	3 (7.5)	6 (20.0)	0.15
Vomiting	9 (12.9)	4 (10.0)	5 (16.7)	0.48
Chills	6 (8.6)	3 (7.5)	3 (10.0)	1.00
Abdominal pain	5 (7.1)	3 (7.5)	2 (6.7)	1.00
Headache	3 (4.3)	3 (7.5)	0 (0.0)	0.25
**Temperature** (°C)				
Mean ± SD	37.32 ± 0.85	37.36 ± 0.96	37.26 ± 0.69	0.61
**Heart rate** (beats/min)				
Mean ± SD	108.44 ± 20.11	104.25 ± 18.81	114.03 ± 20.72	0.04
**Respiratory rate** (breaths/min)				
Mean ± SD	21.60 ± 3.61	20.67 ± 3.46	22.83 ± 3.48	0.01
**SpO2** (%)				
Mean ± SD	90.78 ± 11.79	93.32 ± 6.62	87.40 ± 15.84	0.03
**Body mass index (BMI)**				
Mean ± SD	26.26 ± 7.25	27.47 ± 7.34	24.63 ± 6.91	0.10

SD, standard deviation; SpO2, blood oxygen saturation.

Dyspnea (62.9%), cough (57.1%) and fever (34.3%) were the most common symptoms, followed by muscle ache (31.4%), sore throat (24.3%), diarrhea (12.9%), vomiting (12.9%), chills (8.6%), abdominal pain (7.1%) and headache (4.3%) (**Table 1**). The cancer patients also had other comorbidities, including diabetes (12.9%), hypertension (20.0%) and chronic pulmonary (11.4%), kidney (10.0%), heart (4.3%) and liver (4.3%) diseases (**Table 1**). Non-survivor patients had higher respiratory rates and lower levels of blood oxygen saturation (SpO2) as compared to the survivors. Dyspnea was the most common feature of non- survivor as compared to the survivor (*P*=0.01). No significant differences in age, gender and other comorbidities were detected among survivors and non-survivors groups.

We found significantly elevated levels of D-dimers in the non-survivors as compared to the survivors (*P*=0.01). Furthermore, the non-survivor patients presented with higher levels of neutrophils (*P*=0.01), creatinine (*P*=0.02), blood urea nitrogen (*P*=0.01), lactate (*P*=0.02), aspartate transaminase (*P*=0.02) and ferritin (*P*=0.03) compared to survivor patients. Additionally, C-reactive protein level and neutrophil-lymphocyte ratio (NLR) was higher in the non-survivors than survivors (*P*=0.01), (**Table 2**). 54 (77.14%) out of 70 patients showed abnormal features in the radiological findings (**Supplementary Figure 2**). 23 (32.9%) of 70 patients had ground-glass opacities, and 12(17.1%) presented with consolidation (**Table 2**).

**Table 2:** Laboratory and Radiological findings of patients with cancer and COVID-19.

	Total (n=70)	Alive (n=40, 57.1%)	Dead (n=30, 42.9%)	*P*-value
**White blood cells, ×** 10^3^ cells/µl				
Median (range)	8.41 (0.11-433)	6.73 (0.23-433)	10.68 (0.11-122.82)	0.12
**Neutrophils, ×** 10^3^ cells/µl				
Median (range)	6.69 (1.08-21.52)	4.83 (1.08-21.52)	8.93 (1.70-21.21)	0.01
**Lymphocytes, ×** 10^3^ cells/µL				
Median (range)	1.14 (0.20-116.02)	1.12 (0.20-34.12)	1.15 (0.22-116.02)	0.78
**NLR**				
Median (range)	6.07 (0.05-93.14)	4.49 (0.34-21.10)	7.31 (0.05-93.14)	0.01
**Platelets, ×** 10^3^ cells/µL				
Median (range)	238 (2–852)	256 (20–852)	211 (2–483)	0.27
**RBCs, ×** 10^6^ cells/µL				
Median(range)	4.04 (2.08-11.30)	4.06 (2.37-11.30)	3.81 (2.08-5.54)	0.34
**Hemoglobin,** g/dL				
Median (range)	10.65 (3.97-15)	11.90 (3.97-14.60)	10.10 (5.10-15.0)	0.06
**Creatinine,** mg/dL				
Median (range)	0.66 (0.25-4.67)	0.63 (0.28-1.55)	0.76 (0.25-4.67)	0.02
**Blood urea nitrogen,** mg/dL				
Median (range)	15.04 (3.60-99.14)	12.36 (3.60-37.12)	19.93 (7.49-99.14)	0.01
**Lactate dehydrogenase,** u/L				
Median(range)	380 (172–759)	389.50 (172–759)	365 (194–531)	0.80
**Lactate,** mg/dL				
Median (range)	17.70 (6.80-106.07)	15.00 (6.80-39)	19.65 (9.10-106.07)	0.02
**pH**				
Median(range)	7.47 (7.20-7.54)	7.47 (7.39-7.54)	7.44 (7.20-7.52)	0.17
**PCO2,** mmHg				
Median(range)	34 (17–63)	34.5 (30–49)	34 (17–63)	0.96
**PO2,** mmHg				
Median(range)	76 (31–231)	79 (50–152)	74 (31–231)	0.68
**HCO3,** mmol/L				
Median(range)	25 (10.08-37.50)	25.30 (19.40-31.10)	23.30 (10.08-37.50)	0.20
**O2 saturation,** %				
Median(range)	95 (67–100)	96.50 (88–99)	95 (67–100)	0.54
**ALT,** U/L				
Median(range)	30.50 (9–448)	26 (9–151)	36.50 (15–448)	0.10
**AST,** U/L				
Median(range)	45 (16–1318)	27 (16–288)	53 (21–1318)	0.02
**Albumin,** g/dL				
Median (range)	3.29 (1.70-4.74)	3.52 (1.98-4.74)	2.85 (1.70-3.71)	0.001
**Globulin,** g/dL				
Median(range)	3.25 (1.82-4.50)	3.25 (1.82-4.25)	3.29 (2.00-4.50)	0.98
**GGT,** U/L				
Median(range)	54.50 (13–611)	37.50 (13–434)	58.50 (30–611)	0.07
**Alkaline phosphate,** U/L				
Median(range)	94 (48–870)	93 (48–623)	106.50 (54–870)	0.25
**C-reactive protein,** mg/L				
Median(range)	148 (2-586.43)	94 (4–514)	209.50 (2-586.43)	0.01
**Ferritin,** ng/mL				
Median(range)	915 (63.40-21422)	757(63.40-4056)	1250 (81.20-21422)	0.03
**D-dimer,** mg/L				0.01
<0.2	23 (52.3)	19 (76.0)	4 (21.1)	
≥0.2	21 (47.7)	6 (24.0)	15 (78.9)	
**Radiological findings**				0.07
Ground-glass opacity	23 (32.9)	11 (27.5)	12 (40.0)	
Consolidation	12 (17.1)	4 (10.0)	8 (26.7)	
Patchy shadowing	11 (15.7)	5 (12.5)	6 (20.0)	
Reticulonodular infiltrates	7 (10.0)	6 (15.0)	1 (3.3)	
Others	1 (1.4)	1 (2.5)	0 (0.0)	
Normal	11 (15.7)	9 (22.5)	2 (6.7)	
Unknown	5 (7.1)	4 (10.0)	1 (3.3)	

NLR, neutrophil-lymphocyte ratio; RBCs, red blood cells; ALT, alanine transaminase; AST, aspartate transaminase; GGT, gamma-glutamyl transpeptidase.

Of the 70 patients included, 67 (95.7%) received intravenous antibiotics and 1(1.4%) received antiviral medication (**Table 3**). Montelukast was given to 47 (67.1%) patients. Ivermectin was given to 6 (8.6%) patients. 41 (58.6%) received Zinc and vitamin C. Hydroxychloroquine (HCQ) was given to 4 (5.7%) patients. Tocilizumab was given to 12 (17.1%) patients (**Table 3**). Invasive and non-invasive mechanical ventilation was provided to 19 (27.1%) and 9 (12.9%) patients, respectively. 19 (27.1%) of 70 patients were admitted to the ICU. 51(72.9%) of 70 patients developed the complications. Additionally, 30 (58.82%) of 51 patients having complications were non-survivors. We observed that non-survivors were more likely to receive oxygen therapy, required mechanical ventilation, were referred to the ICU, where they developed complications such as ARDS 32 (45.7%), acute renal failure 23 (32.9%), septic shock 22 (31.4%), abnormal liver function 25 (35.7%), coagulopathy16 (22.9%), secondary infections 26 (37.1%) and arrhythmia 8 (11.4%), (**Table 3**).

**Table 3:** Treatments and complications of patients with cancer and COVID-19.

	Total(n=70)	Alive (n=40, 57.1%)	Dead (n=30, 42.9%)	*P*-value
**Treatments**				
Intravenous antibiotic	67 (95.7)	37 (92.5)	30 (100.0)	0.25
Antiviral medication	1 (1.4)	1 (2.5)	0 (0.0)	1.00
Tocilizumab	12 (17.1)	4 (10.0)	8 (26.7)	0.06
Montelukast	47 (67.1)	29 (72.5)	18 (60.0)	0.27
Ivermectin	6 (8.6)	0 (0.0)	6 (20.0)	0.05
Zinc (vitamin C)	41 (58.6)	25 (62.5)	16 (53.3)	0.44
HCQ	4 (5.7)	2 (5.0)	2 (6.7)	1.00
Oxygen therapy	61 (87.1)	31 (77.5)	30(100.0)	0.01
**Mechanical ventilator**				
None	42 (60.0)	35 (87.5)	7 (23.3)	0.001
Non-invasive	9 (12.9)	3 (7.5)	6 (20.0)	
Invasive	19 (27.1)	2 (5.0)	17 (56.7)	
**ICU admission**	19 (27.1)	4 (10.0)	15 (50.0)	0.001
CRRT	9 (12.9)	0 (0.0)	9 (90.0)	0.001
**Complications**	51 (72.9)	21 (52.5)	30 (100.0)	0.001
ARDS	32 (45.7)	6 (15.0)	26 (86.7)	0.001
Acute renal failure	23 (32.9)	2 (5.0)	21 (70.0)	0.001
Septic shock	22 (31.4)	3 (7.5)	19 (63.3)	0.001
Abnormal liver function	25 (35.7)	9 (22.5)	16 (53.3)	0.001
Coagulopathy	16 (22.9)	1 (2.5)	15 (50.0)	0.001
Secondary infection	26 (37.1)	7 (17.5)	19 (63.3)	0.001
Arrhythmia	8 (11.4)	1 (2.5)	7 (23.3)	0.01
Other complications	22 (31.4)	11 (27.5)	11 (36.7)	0.41

HCQ, hydroxychloroquine; ICU, Intensive-care unit; CRRT, continuous renal replacement therapy; ARDS, acute respiratory distress syndrome.

**Figure 1:**
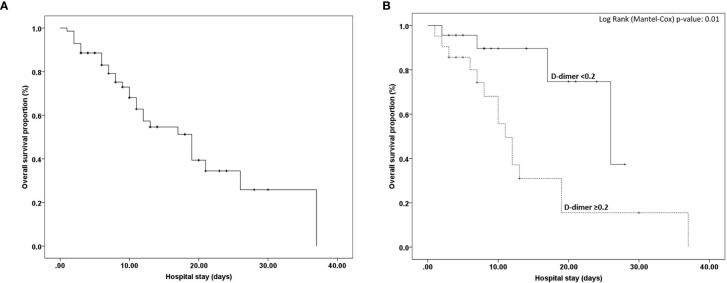
**(A)** Overall Survival of COVID-19 cancer patients **(B)** Overall Survival of COVID-19 cancer patients stratified by two groups of D-dimer (<0.2 and ≥0.2).

57 (81.4%) of 70 patients were diagnosed with solid tumors and 13 (18.6%) of 70 patients were diagnosed with hematological malignancies (**Table 4**). The most common cancers were in breast 11 (19.3%), prostate 5 (8.8%), colorectal 4 (7.0%), lung 4 (7.0%), kidney 3 (5.3%) and oral 3 (5.3%). Among hematological malignancies lymphoma (5cases:38.5%) was most common. 53 (75.7%) of 70 patients received the chemotherapy. Radiotherapy was given to 33 (47.1%) of 70 patients. 25 (35.7%) of 70 patients received chemotherapy within 4 weeks before the onset of symptoms. 23 (32.9%) of 70 patients were diagnosed with cancer within the past year and 23 (32.9%) of 70 patients had an ECOG score higher than the one before admission. 44 (72.1%) of 70 patients were with an advanced stage (III-IV). The case fatality rate in patients with solid tumors was 40.3% (23 of 57 patients) and that in hematological malignancies was 53.8% (7 of 13 patients), (**Table 4**). The median time of hospital stay was 8 days (range 1-37 days). The overall median survival time of hospital stay was 19 days (confidence interval 13-25) **Figure 1A**. Overall survival was compared among the D-dimer categories, <0.2 and ≥0.2. There was a statistically significant difference (*P*=0.01) between the two groups (**Figure 1B**). The median survival time in D-dimer was lowest in ≥0.2 category (11 days with confidence interval 8-13) compared to D-dimer <0.2 category (26 days with confidence interval 13-39). Overall survival of D-dimer ≥0.2 category was significantly associated with mortality (*P*-value 0.01) as shown in **Figure 1B**.

**Table 4:** History of cancer in patients with COVID-19.

	Total(n=70)	Alive(n=40, 57.1%)	Dead(n=30, 42.9%)	*P*-value
**Cancer Type**				0.37
**Hematological malignancy**	13 (18.6)	6 (15.0)	7 (23.3)	
Acute lymphoblastic leukemia	3 (23.1)	3 (50.0)	0 (0.0)	
Chronic lymphocytic leukemia	2 (15.4)	1 (16.7)	1 (14.3)	
Chronic myeloid leukemia	1 (7.7)	0 (0.0)	1 (14.3)	
Leukemia	1 (7.7)	0 (0.0)	1 (14.3)	
Lymphoma	5 (38.5)	2 (33.3)	3 (42.9)	
Multiple myeloma	1 (7.7)	0 (0.0)	1 (14.3)	
**Solid tumor**	57 (81.4)	34 (85.0)	23 (76.7)	
Breast	11 (19.3)	7 (20.6)	4 (17.4)	
Prostate	5 (8.8)	4 (11.8)	1 (4.3)	
Colorectal	4 (7.0)	1 (2.9)	3 (13.0)	
Lung	4 (7.0)	1 (2.9)	3 (13.0)	
Kidney	3 (5.3)	1 (2.9)	2 (8.7)	
Oral	3 (5.3)	2 (5.9)	1 (4.3)	
Others	27 (47.4)	18 (52.9)	9 (39.1)	
**History of treatments**	62 (88.6)	38 (95.0)	24 (80.0)	0.06
Surgery	40 (57.1)	26 (65.0)	14 (46.7)	0.12
Chemotherapy	53 (75.7)	31 (77.5)	22 (73.3)	0.68
Radiotherapy	33 (47.1)	22 (55.0)	11 (36.7)	0.12
**Chemotherapy within 4 weeks before symptoms onset**				
Chemotherapy	25 (35.7)	15 (37.5)	10 (33.3)	0.71
**Cancer stage**				0.19
Early (I-II)	17 (27.9)	12 (34.3)	5 (19.2)	
Advance (III-IV)	44 (72.1)	23 (65.7)	21 (80.8)	
**Metastasis**	23 (32.9)	15 (37.5)	8 (26.7)	0.34
**Time since cancer diagnosis**				0.23
<1	23 (32.9)	15 (37.5)	8 (26.7)	
1-5 years	36 (51.4)	17 (42.5)	19 (63.3)	
>5	11 (15.7)	8 (20.0)	3 (10.0)	
**ECOG performance status score**				0.35
0	13 (18.6)	10 (25.0)	3 (10.0)	
1	23 (32.9)	13 (32.5)	10 (33.3)	
2	27 (38.6)	15 (37.5)	12 (40.0)	
3	3 (4.3)	1 (2.5)	2 (6.7)	
4	4 (5.7)	1 (2.5)	3 (10.0)	

ECOG, Eastern Cooperative Oncology Group.

In a univariable logistic regression model, dyspnea, respiratory rate, heart rate, ICU admission, creatinine, neutrophil, albumin, blood urea nitrogen, C-reactive protein and D-dimer values were associated with death. In addition, SpO2 and lactate were marginally statistically significant with respect to death in the univariable logistic regression model (**Table 5**). In a multivariable logistics regression model, we identified two variables as independent risk factors of death, ICU admission (odds ratio (OR), 21.79; 95% confidence interval (CI), 2.31–205.68, *P*=0.007) and D-dimer values (OR, 26.67; 95% CI, 2.97-239.30, *P*=0.003).

**Table 5:** Adjusted logistic regression analysis of factors associated with mortality.

Variables	Categories	Univariable Odds ratio (confidence interval), *P*-value	Multivariable Odds ratio(confidence interval), *P*-value
**Age (years)**	Mean ± SD	1.02 (1.00-1.05), 0.18	–
**Sex**	Male	Ref	–
	Female	0.52 (0.19-1.37), 0.19	–
**BMI**	Mean ± SD	0.94 (0.87-1.01), 0.11	–
**Dyspnea**	No	Ref	–
	Yes	4.00 (1.34-11.87), 0.01	–
**Respiratory rate** (breaths/min)	Mean ± SD	1.24 (1.02-1.50), 0.02	–
**SpO2** (%)	Mean ± SD	0.95 (0.90-1.00), 0.06	–
**Heart rate** (beats/min)	Mean ± SD	1.02 (1.00-1.05), 0.04	–
**Comorbidity**	No	Ref	–
	Yes	0.96 (0.36-2.57), 0.94	–
**Cancer stage**	Early	Ref	–
	Advance	2.19 (0.66-7.27), 0.20	–
**ICU admission**	No	Ref	Ref
	Yes	9.00 (2.56-31.24), 0.001	21.79 (2.31-205.68), 0.007
**Creatinine,** mg/dL	Mean ± SD	4.31 (1.20-15.38), 0.02	–
**Lactate,** mg/dL	Mean ± SD	1.05 (1.00-1.13), 0.08	–
**Alkaline phosphate,** U/L	Mean ± SD	1.00 (1.00-1.00), 0.26	–
**Neutrophil, ×** 10^3^ cells/µl	Mean ± SD	1.14 (1.03-1.28), 0.01	–
**Hemoglobin,** g/dL	Mean ± SD	0.83 (0.67-1.02), 0.08	–
**Albumin,** g/dL	Mean ± SD	0.10 (0.02-0.40), 0.002	–
**Blood urea nitrogen,** mg/dL	Mean ± SD	1.08 (1.02-1.15), 0.006	–
**C-reactive protein,** mg/L	Mean ± SD	1.01 (1.00-1.09), 0.01	–
**D-dimer,** mg/L	<0.2	Ref	Ref
	≥0.2	11.87 (2.83-11.87), 0.001	26.67 (2.97-239.30), 0.003

BMI, body mass index; SpO2, blood oxygen saturation; ICU, intensive-care unit.

## Discussion

Cancer patients are considered as highly vulnerable individuals in the COVID-19 pandemic (7–11). These individuals are at high risk to develop serious illness once diagnosed with COVID-19 infection. The mortality rate is high as well in these individuals (7). In addition to the few studies recently reported from China, this is the first retrospective study conducted in Pakistan to illustrate the comparative analysis of clinical characteristics, treatment, radiological and laboratory findings of survivor and non-survivor COVID-19-infected cancer patients. In particular, we identified two variables as independent risk factors of death: ICU admission and D-dimer.

SARS-CoV-2 infection may lead to acute respiratory distress syndrome (18). Dyspnea (62.9%), cough (57.1%) and fever (34.3%) are the most common symptoms as described in previous studies (7, 19). Liang et al. observed that old age and comorbidities were the major risk factors (13). This was not observed in our study and our results were similar to the study published by Yang et al. (7). We further observed abnormal respiratory rate, heart rate and blood oxygen saturation in the COVID-19-infected cancer patients. A retrospective study reported a link between hypoxemia (SpO2 <90%) and mortality in COVID-19 patients (20). We found a significant difference in SpO2 in survivors and non-survivors patients. Yang et al. observed higher respiratory rates and lower levels of blood oxygen saturation among non-survivors (7). Our results are in keeping with this study, as we also found a significant difference in respiratory rates in survivors and non-survivors COVID-19 infected cancer patients.

In addition to previously reported data, D-dimers are usually elevated in patients with COVID-19 (21). Yao et al. observed an association of increased D-dimer level (> 2.14 mg/L) with in-hospital mortality (21). Tian and colleagues showed that D-dimer might play a predictive role for COVID-19 severity particularly in cancer patients (22). Yang et al. also observed elevated level of D-dimer in non-survivors as compared to survivors cancer patients with COVID-19 (7). We also found that higher levels of D-dimer (≥0.2 mg/L) were significantly different between non-survivor and survivor cancer patients infected with COVID-19. Additionally, D-dimers ≥0.2 was significantly associated with mortality. In this regard, the first post-mortem analysis of COVID-19 microscopic lesions reported by Varga et al. showed direct viral involvement of endothelial structures (endothelialitis) (23), a phenomenon which may be linked with the coagulopathy (increase in D-dimers) observed in this study. These findings may justify the use of anti-viral or anti-COVID 19 antibody treatments to ameliorate the outcome of COVID-19-positive cancer patients.

Inflammatory cytokines are involved in the severity and adverse clinical outcomes in COVID-19 infected patients (24). Zhang et al. reported high neutrophil counts and C-reactive protein levels in cancer patients infected with COVID-19 (24). We also observed higher neutrophil counts and C-reactive protein levels in non-survivor *vs* survivor cancer patients infected with COVID-19. There is a possibility that pro-inflammatory neutrophils and C-reactive protein induce “cytokine storm” related to endothelialitis caused by COVID-19 infected cancer patients (23, 24). Serum lactate is used to assess the disease severity, therapeutic response and disease outcomes (25). Evaluating the lactate level has clinical significance in the management of critical cases of severe sepsis (26). Previously published data have shown that hyperlactatemia may be a predictor of death (27). In our results, we found a significant increase in the lactate levels of non-survivor COVID-19 infected cancer patients. As COVID-19 disease severity changes rapidly, it is beneficial to identify the parameters of severity at initial stage. Several studies also identified higher levels of creatinine (7, 28), blood urea nitrogen (7, 28), aspartate transaminase (29, 30), and ferritin (31), that are associated with the severity of COVID-19 infection. We also found elevated levels of creatinine, blood urea nitrogen, aspartate transaminase, and ferritin in non-survivor COVID-19 infected cancer patients.

Xu et al. reported bilateral lung lesions in 53 (59%) of 90 patients (32). We found bilateral lung lesions in 70% of our data set, which was higher than reported previously (32). The involvement of the lungs suggests that cancer patients are more prone to severe pulmonary disease after COVID-19-infection. Furthermore, we observed that non-survivors were significantly more likely to receive oxygen therapy, require mechanical ventilation, and be referred to the ICU. Complications such as ARDS, acute renal failure, septic shock, coagulopathy, secondary infections and arrhythmia were common in these patients. Our results are in keeping with the data published previously (7). Yang et al. reported that COVID-19 cancer patients receiving chemotherapy within 4 weeks before the onset of symptoms were more prone to death during hospital admission (7). In contrast, Lee et al. conducted a study on COVID-19 cancer patients and reported that chemotherapy had no effect on patient mortality (33). We found similar results with our limited retrospective cohort of COVID-19 infected cancer patients who received chemotherapy within 4 weeks before the onset of symptoms, which made no significant difference in mortality as compared to those who did not receive it. Clearly, a long-term follow up of the COVID-19 infected survivor cancer patients is required to identify the impact of the disease. The current study is a retrospective cohort, in which we included only those cancer patients who were admitted to our hospital from April 13 to July 9, 2020. The sample size of our study was limited because we could not include all cancer patients who tested positive for COVID-19infection since the clinical and pathological data was available for admitted cancer patients only. In addition, we have not evaluated the alteration in hematological parameters during hospitalization. Another limitation of our study is that we could not include non-cancer patients since our hospital is a specialized cancer care hospital.

In conclusion, we provide comprehensive information of the clinical characteristics, laboratory data and outcomes of COVID-19-infected cancer patients from Pakistan. This study highlights that elevated D-dimer levels and ICU admission as the two major independent risk factors for early surveillance of disease progression. Furthermore, we need to be more vigilant to the levels of C-reactive protein and neutrophils, once cancer patients are infected with COVID-19. The data presented in this study will certainly serve as a useful addition to the already available knowledge and to decide whether an anti-viral treatment is required or not. The risk factors might help clinicians to determine who are the high-risk cancer patients at the early onset of the infection.

## Data Availability Statement

The original contributions presented in the study are included in the article/**Supplementary Material**. Further inquiries can be directed to the corresponding author.

## Ethics Statement

The studies involving human participants were reviewed and approved by Shaukat Khanum Memorial Cancer Hospital and Research Centre - Institutional Review Board (IRB). Written informed consent for participation was not required for this study in accordance with the national legislation and the institutional requirements.

## Author Contributions

KA had the idea for and designed the study. KA, MA, MJA, AF, KS, IR, and JA were involved in the acquisition of the data. KA, MA, and MJA summarized the data. KA, MA, MJA, IR, JA, and KS were involved in data interpretation. KA drafted the manuscript. MA, KS, IR, JA, MR, AK, and AL critically revised the manuscript for important intellectual content. All authors contributed to the article and approved the submitted version.

## Conflict of Interest

The authors declare that the research was conducted in the absence of any commercial or financial relationships that could be construed as a potential conflict of interest.
