# Supplementary material for: Clinical Characteristics of COVID-19-Infected Cancer Patients in Pakistan: Differences Between Survivors and Non-Survivors
Source: Front Oncol. 2021 May 20;11:655634. doi: 10.3389/fonc.2021.655634 (PMC8173078; doi:10.3389/fonc.2021.655634)
Supplement: Supplementary file 1 [file DataSheet_1.docx]

13,692 patients diagnosed with COVID-19 in electronic medical record system from Shaukat Khanum Memorial Cancer Hospital and Research Centre (SKMCH&RC), Pakistan, between April 13 and July 09, 2020

443 cancer patients with COVID-19 infection in electronic medical record system

362 cancer patients excluded, as they were not hospitalized

81cancer patients with COVID-19 symptoms admitted at SKMCH&RC

11 patients excluded, as they were under the age of 18 years

70 patients with a history of solid or hematological malignancies included in the study

**Figure-1** Study population


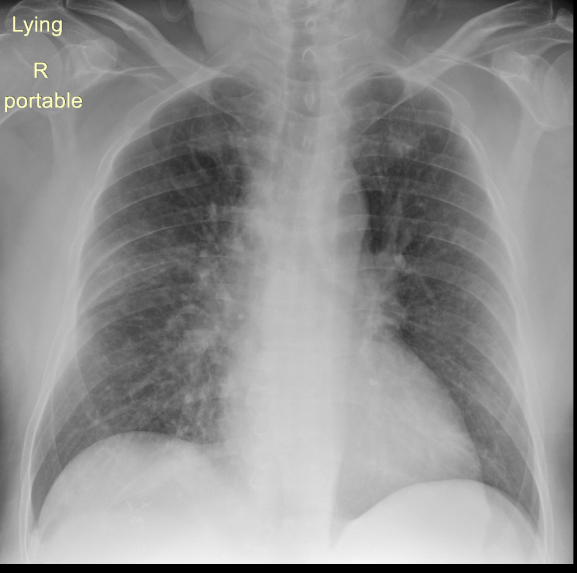

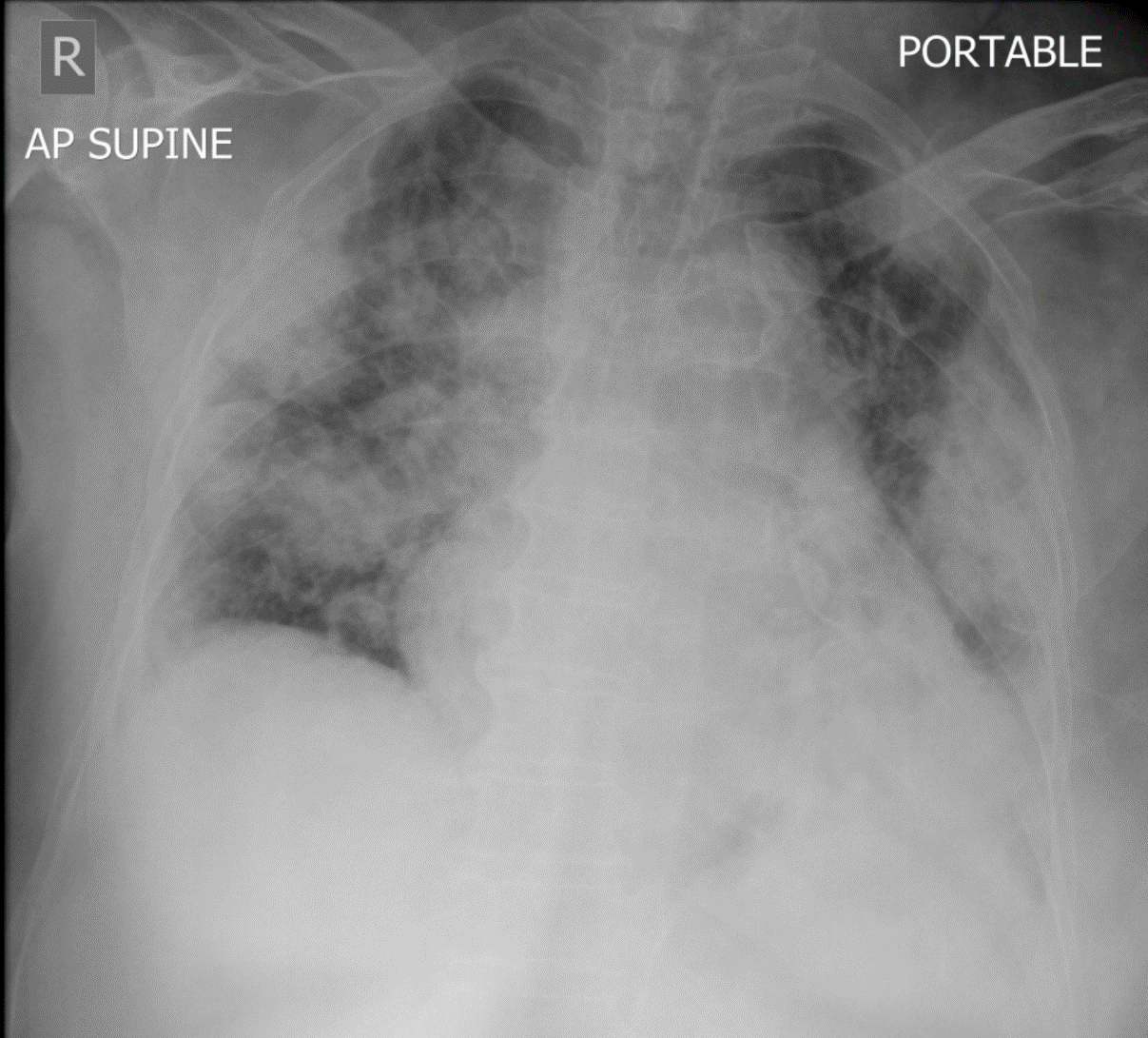


A

B


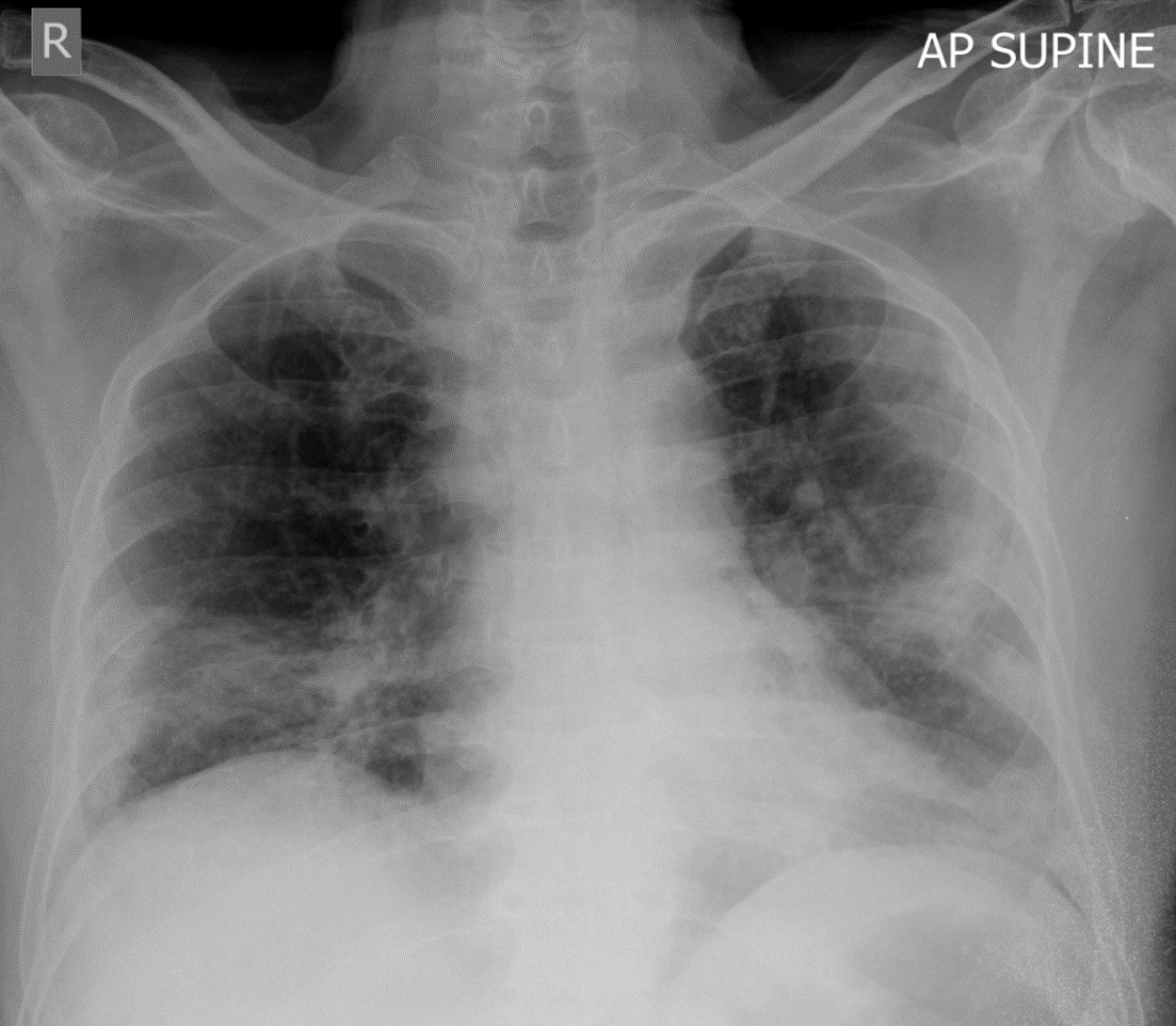

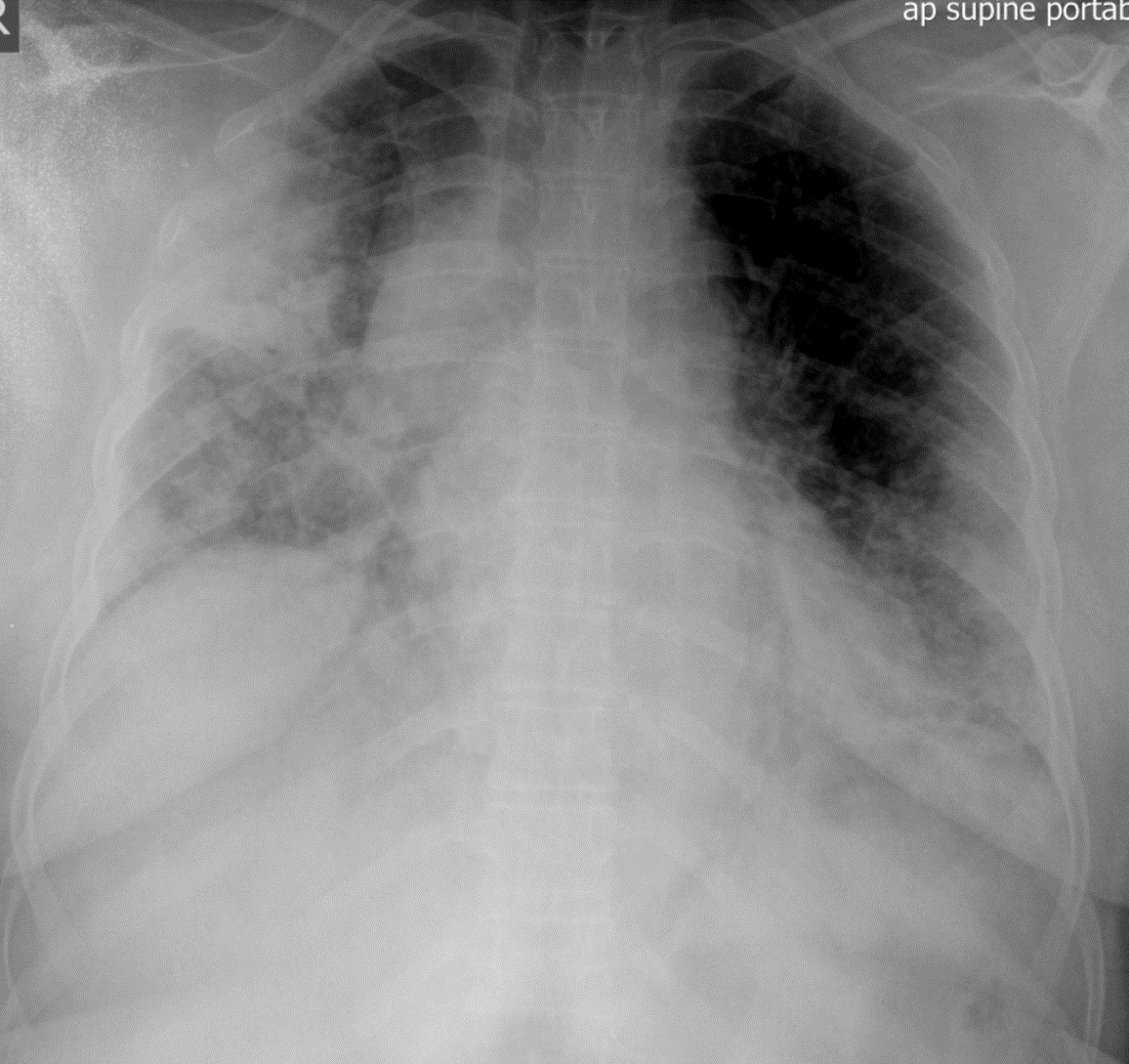


D

C

**Figure-2: (A)** Chest X-ray image of 66 year-old male patient with coronavirus disease (COVID-19) taken on June 8,2020, showing patchy opacities in the right lung with pneumonia. **(B)** Chest X-ray image of a 64-year-old female patient with COVID-19 taken on June 16, 2020, showing interval development of diffuse bilateral airspace disease, which is compatible with COVID pneumonia and consolidation. **(C)** Chest X-ray image of a 63-year-old male patient with COVID-19 taken on June 15, 2020, showing interval development of patchy air space disease in the right mid and left lower and retrocardiac zone, furthermore it is compatible with COVID pneumonia. **(D)** Chest X-ray image of a 56-year-old female patient with COVID-19 taken on June 9, 2020, showing bilateral peripheral reticulonodular infiltrates with consolidation.
